# Metformin increases survival in hormone receptor-positive, HER2-positive breast cancer patients with diabetes

**DOI:** 10.1186/s13058-015-0574-3

**Published:** 2015-05-03

**Authors:** Hee Jeong Kim, Hyunwook Kwon, Jong Won Lee, Hwa Jung Kim, Sae Byul Lee, Hee Sung Park, Guiyun Sohn, Yura Lee, Beom Seok Koh, Jong Han Yu, Byung Ho Son, Sei Hyun Ahn

**Affiliations:** Division of Breast and Endocrine Surgery, Department of Surgery, Asan Medical Center, University of Ulsan College of Medicine, Seoul, Korea; Division of Vascular Surgery, Department of Surgery, Asan Medical Center, University of Ulsan College of Medicine, Seoul, Korea; Department of Clinical Epidemiology and Biostatics, Asan Medical Center, University of Ulsan College of Medicine, Seoul, Korea

## Abstract

**Introduction:**

Metformin use has recently been observed to decrease both the rate and mortality of breast cancer. Our study was aim to determine whether metformin use is associated with survival in diabetic breast cancer patients by breast cancer subtype and systemic treatment.

**Methods:**

Data from the Asan Medical Center Breast Cancer Database from 1997 to 2007 were analyzed. The study cohort comprised 6,967 nondiabetic patients, 202 diabetic patients treated with metformin, and 184 diabetic patients that did not receive metformin. Patients who were divided into three groups by diabetes status and metformin use were also divided into four subgroups by hormone receptor and HER2-neu status.

**Results:**

In Kaplan-Meier analysis, the metformin group had a significantly better overall and cancer specific survival outcome compared with non metformin diabetic group (*P* <0.005 for both). There was no difference in survival between the nondiabetic and metformin groups. In multivariate analysis, Compared with metformin group, patients who did not receive metformin tended to have a higher risk of metastasis with HR 5.37 (95 % CI, 1.88 to 15.28) and breast cancer death with HR 6.51 (95 % CI, 1.88 to 15.28) on the hormone receptor-positive and HER2-negative breast cancer. The significant survival benefit of metformin observed in diabetic patients who received chemotherapy and endocrine therapy (HR for disease free survival 2.14; 95 % CI 1.14 to 4.04) was not seen in diabetic patients who did not receive these treatments.

**Conclusion:**

Patients receiving metformin treatment when breast cancer diagnosis show a better prognosis only if they have hormone receptor-positive, HER2-positive tumors. Metformin treatment might provide a survival benefit when added to systemic therapy in diabetic patients.

## Introduction

Diabetes has been found to be a risk factor for breast cancer in some studies, and patients with diabetes may have poorer outcomes than nondiabetic patients [1– 3]. A recent British cohort study found that women with breast cancer and pre-existing diabetes had a 49 % (95 % CI, 1.17 to 1.88) increased all-cause mortality risk compared with women with breast cancer but without diabetes [4]. Obesity is associated with type 2 diabetes and is itself a risk factor for breast cancer and possibly for a poorer outcome from this cancer [5]. The common factor linking diabetes, obesity, and metabolic syndrome to cancer may be insulin resistance and the consequent hyperinsulinemia associated with these conditions [6].

Metformin is widely used in the treatment of type 2 diabetes mellitus to increase insulin sensitivity and improve glycemic control [7, 8]. In addition, numerous experimental, epidemiologic, observational, and clinical studies have shown that metformin has antitumor effects [9, 10]. In particular, metformin treatment has been associated with lower breast cancer incidence among patients with diabetes and higher pathologic complete response in patients with early-stage breast cancer who were receiving neoadjuvant therapy [11]. Metformin could decrease breast cancer cell growth either indirectly by reducing circulating insulin and insulin-like growth factor (IGF) or directly via activation of adenosine monophosphate-activated protein kinase (AMPK) [12]. Initiation of an AMPK-dependent energy stress response, resulting in inhibition of the mammalian target of rapamycin (mTOR) signaling pathway, leads to reduced protein synthesis and proliferation of cancer cells [13]. Thus, the anticancer effects of metformin are mediated through a systemic improvement in the metabolic profile and by directly targeting tumor cells [10]. However, results from observation studies on survival benefit in breast cancer patients with diabetes are conflicting. A recent study failed to show a significant reduction in breast cancer mortality in patients treated with metformin [14] but some fairly small studies have reported a beneficial effect of metformin use on survival in patients with human epidermal growth factor receptor-2 (HER2)-positive tumors [15].

Based on the evidence reviewed above, a growing number of clinical intervention studies of metformin have been initiated [16]. A phase III trial of adjuvant metformin has been initiated in women with breast cancer (NCIC CTC MA.32) [9] and a phase II trial of a neoadjuvant setting for postmenopausal breast cancer patients (METEOR study) has also started patient enrolment [10]. However, the accrual and treatment process is still ongoing, and several years of follow up are needed to determine if there is any survival benefit. In addition, little is known about the effects of metformin on different subtypes of breast cancer. In our current study, we explored the association between metformin use and survival outcomes in diabetic and nondiabetic breast cancer patients and analyzed benefit according to breast cancer subtype using immunohistochemical staining.

## Methods

This study was approved by the institutional review board of Asan medical center and conducted in accordance with the Helsinki declaration. No consent was needed for this retrospective analysis using a web-based database without personal information.

### Patients

Data from the Asan Medical Center Breast Cancer Database (AMCBCD) from 1997 to 2007 were analyzed. A total of 6,967 patients who were diagnosed with breast cancer and underwent surgery were included in this study. Patients were categorized by diabetes status and metformin use. They are not preselected on these exposures. Patients with a database diagnostic code (ICD-9, 250) during their outpatient clinic visits or a hospital admission were assigned to the diabetic group. For the diabetic patients, whether or not they received metformin as an antidiabetic drug on diagnosis of breast cancer was the criterion for dividing the groups into metformin or non-metformin users.

AMCBCD provides detailed clinicopathologic information on breast cancer. Eight surgical oncologists who specialized in breast cancer reviewed all available survival data for this study. Patient age (<50 years versus ≥50 years), body mass index (BMI), tumor size, lymph node involvement, hormone receptor and HER2-neu status, and adjuvant treatment status were recorded. Survival data, which included disease-free survival (DFS), cancer-specific survival (CSS) and overall survival (OS) were included in this cohort, and for the survival data, eight breast cancer specialists checked the survival data using patients’ records. Positive staining for estrogen receptor (ER) or progesterone receptor (PR) was defined as a score of more than 3+ and HER2 positivity was defined as a score of 3+ by immunohistochemical (IHC) staining or *HER2* gene amplification by fluorescence *in situ* hybridization. The hormone receptor-positive group comprised ER-positive and/or PR-positive patients. Hormone therapy included adjuvant tamoxifen, ovarian suppression or ablation with endocrine therapy, and aromatase inhibitors. Until 2007, the anti-HER2 therapy (Trastuzumab) was not covered by Korean government health insurance. Few people had received anti-HER2 treatment among the patients who had HER2/neu-overexpressing breast cancer. The patients who had received anti-HER2 treatment were excluded from this study. Patients were divided into three groups by diabetes status and metformin use: a nondiabetic group, a metformin group (diabetic patients who received metformin), and a non-metformin group (diabetic patients who did not receive metformin after diagnosis of breast cancer). Moreover, patients were also divided into four subgroups by hormone receptor and HER2-neu status: hormone receptor-positive and HER2-negative, hormone receptor-positive and HER2-positive, hormone receptor-negative and HER2-positive, and hormone receptor-negative and HER2-negative.

### Statistical analysis

**Fig. 1:**
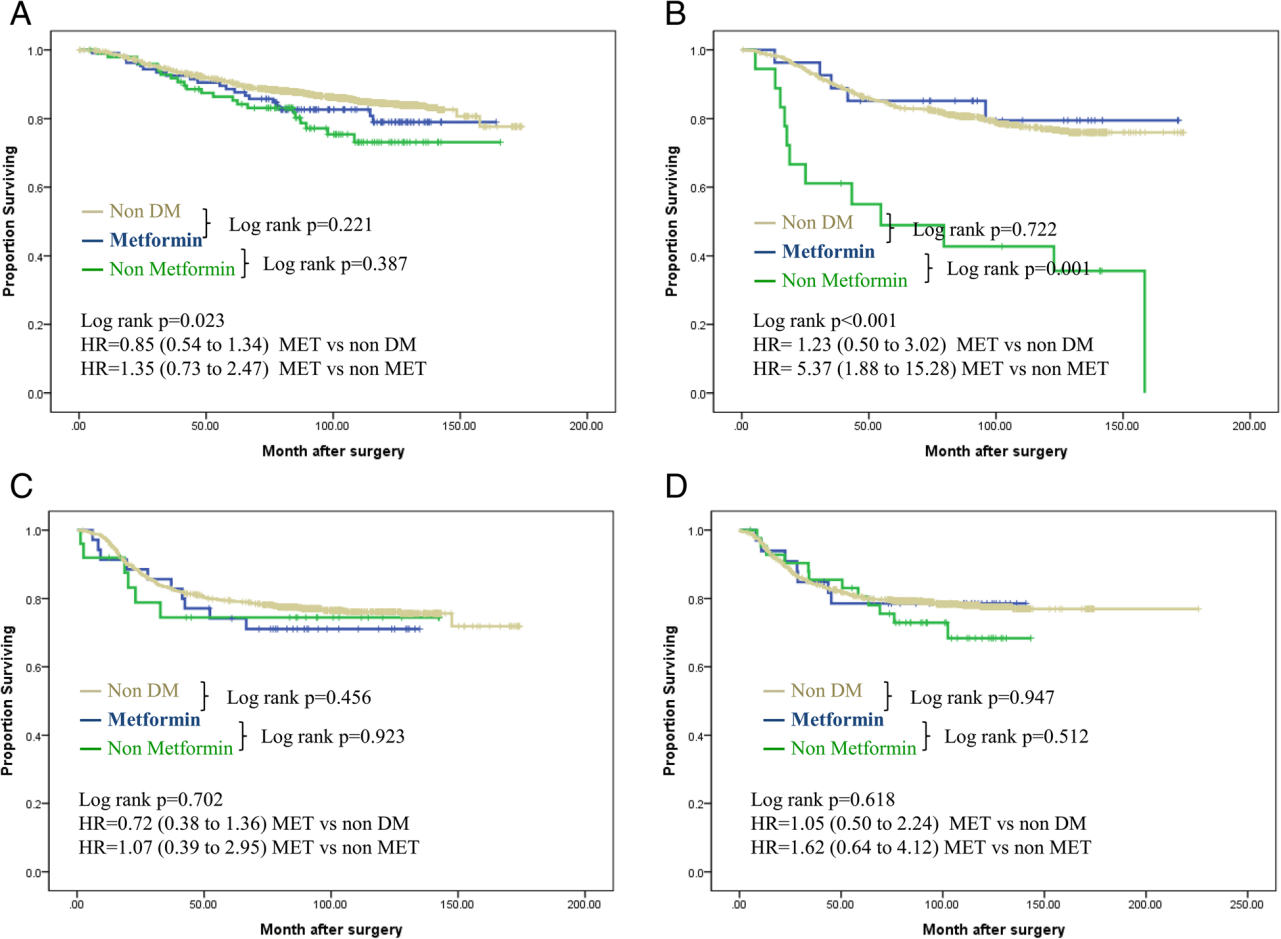
Disease-free survival according to diabetes mellitus and metformin treatment among different intrinsic subtypes using immunohistochemical staining of estrogen receptor (ER), progesterone receptor (PR) and human epidermal growth factor receptor-2 (HER2). **(a)** Hormone receptor-positive, HER2-negative. **(b)** Hormone receptor-positive, HER2-positive. **(c)** Hormone receptor-negative, HER2- positive. **(d)** Hormone receptor-negative, HER2-negative Adjusted for tumor size (≤2 versus > 2 cm), lymph node status (positive versus negative), ER status, PR status, and HER2-neu status (non-amplification versus amplification). HR, hazard ratio; MET, metformin; DM, diabetes mellitus

**Fig. 2:**
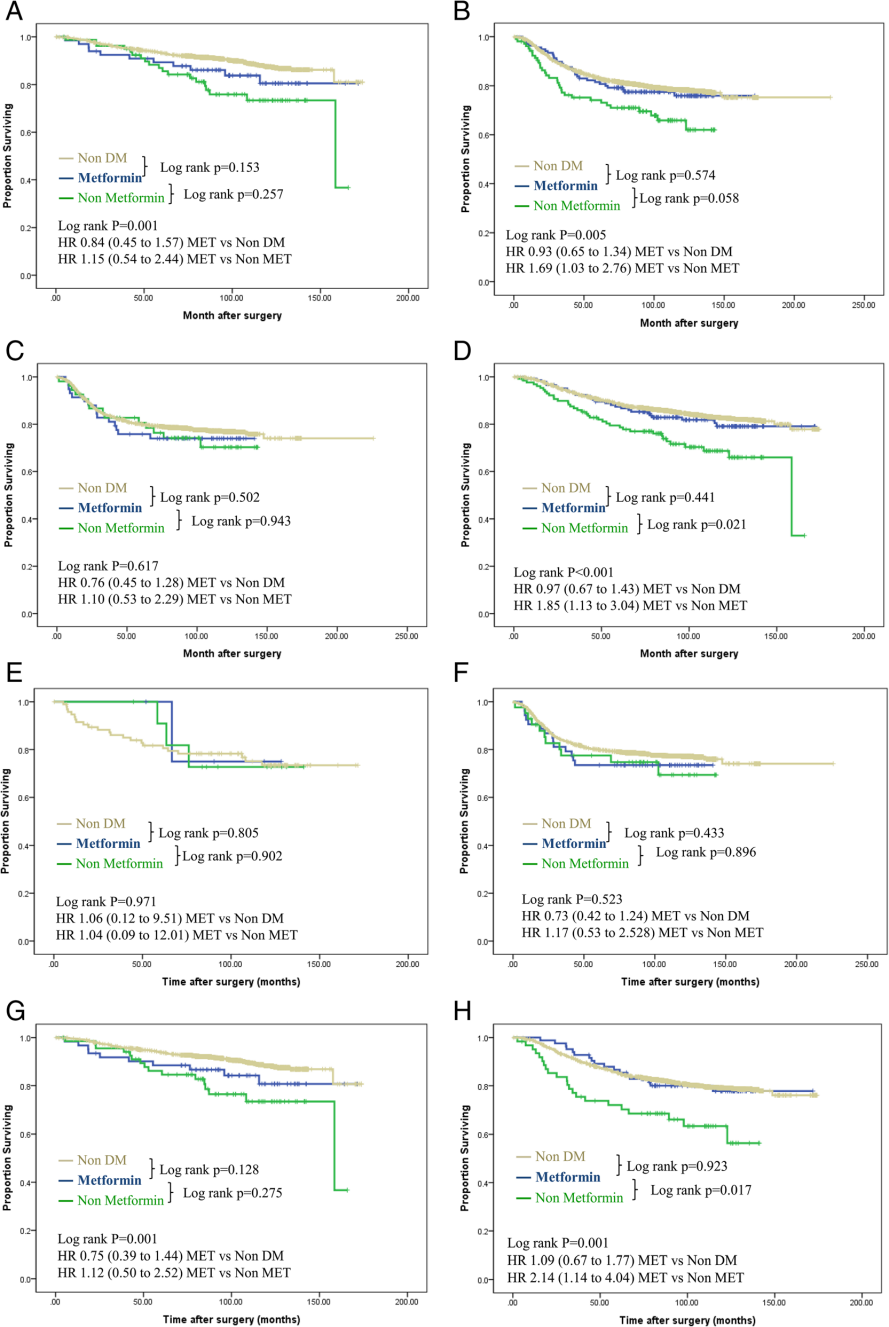
Disease-free survival according to systemic treatment of breast cancer among nondiabetic patients, diabetic patients receiving metformin, and diabetic patients not receiving metformin. **(a)** Patients who received chemotherapy. **(b)**. Patients who did not receive chemotherapy. **(c)** Patients who received endocrine therapy. **(d)** Patients who did not received endocrine therapy. **(e)** Patients who did not receive any treatment. **(f)** Patients who received only chemotherapy. **(g)** Patients who received endocrine therapy only. **(h)** Patients who received chemotherapy and endocrine therapy. Adjusted for tumor size (≤2 versus > 2 cm), lymph node status (positive versus negative), estrogen receptor status, progesterone receptor status, and human epidermal growth factor receptor-2 (HER2)-neu. status (non-amplification versus amplification). HR, hazard ratio; MET, metformin; DM, diabetes mellitus

**Fig. 3:**
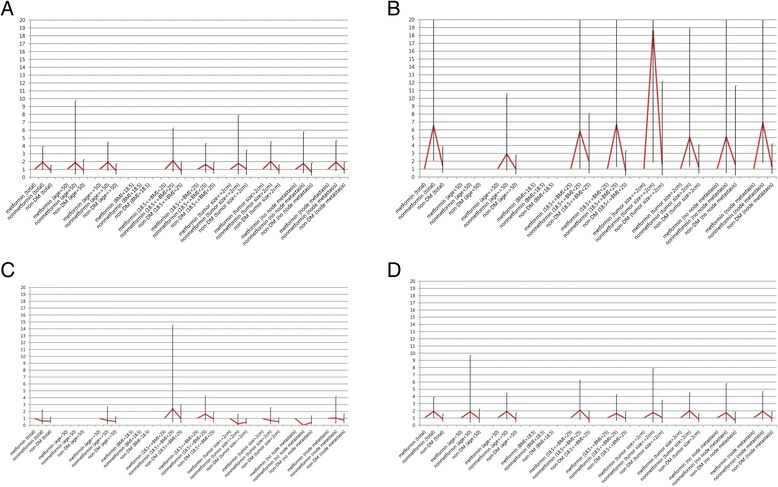
Recurrence risk of breast cancer according to each prognostic factor. **(a)** Hormone receptor-positive, human epidermal growth factor receptor-2 (HER2)-negative. **(b)** Hormone receptor-positive, HER2-positive. **(c)** Hormone receptor-negative, HER2-positive. **(d)** Hormone receptor-negative, HER2-negative

The demographic and clinical characteristics of the three study groups were analyzed with the chi-square test and the differences among groups were compared with one-way analysis of variance (ANOVA) and post hoc Scheffé test. The primary endpoint was DFS. Events used for the analysis of the end point of disease-free survival included local, regional, and contralateral breast cancer or distant breast cancer recurrence. Curves for DFS (until date of recurrence or death), CSS (until date of death from breast cancer), OS (until date of death) were calculated with the Kaplan-Meier product-limit method. Differences between groups were analyzed with the log-rank test. The relationships of risk factors (age, BMI, tumor size, lymph node metastasis, ER status, PR status, HER2-neu status, chemotherapy and hormone therapy) to disease-free survival, cancer-specific survival and OS were analyzed with multivariate Cox regression analysis. For subgroup analysis using IHC (Fig. 1, Table 1) and systemic treatment (Fig. 2), chemotherapy and hormonal therapy treatment were not considered for multivariate analysis. On multivariate analysis, the metformin group was regarded as a reference for calculating the hazard ratio (HR). To exclude a biased result, sensitivity analysis was conducted to check the consistency of the HR according to each clinically important prognostic factor for breast cancer recurrence (Fig. 3). All other statistical analyses were carried out with SPSS software (version 18.0; SPSS, Chicago, IL, USA), with a *P*-value ≤0.05 considered statistically significant.

**Table 1:** Patient demographics and clinical characteristics by study group

	Metformin		Non metformin		Non diabetic		*P*-value
	(n = 202)		(n = 184)		(n = 6,581)		
	Number	%	Number	%	Number	%	
**Age at operation, years**							
Median (range)	55(10.5)		59(10.2)		46(9.6)		<0.001
<50	68	33.7	40	21.7	4370	66.4	
≥50	134	66.3	144	78.3	2211	33.6	
**Body mass index, kg/m** ^**2**^							
<18.5	0	0	4	2.2	242	3.7	<0.001
≥18.5, <25	84	41.6	75	40.8	4644	70.8	
≥25	118	58.4	105	57.1	1669	25.5	
**Serum glucose, mg/dL**							
Median	165.5		150.5				
≤150	83	40.7	93	50			0.068
>150	121	59.3	93	50			
**HbA1C, %**							
<7	51	32.1	29	35.8			0.566
≥0.5108	67.9	52	64.2				
**Tumor size, cm**							
≤2	100	49.5	93	50.5	3535	53.7	0.356
>2	102	50.5	91	49.5	3046	46.3	
**Node metastasis**							
No	110	54.5	110	59.8	3860	58.7	0.463
Yes	92	45.5	74	40.2	2721	41.3	
**Chemotherapy**							
No	66	32.7	80	43.5	1799	27.3	<0.001
Yes	136	67.3	104	56.5	4782	72.7	
**Endocrine therapy**							
No	58	28.7	54	29.3	2013	30.6	0.801
Yes	144	71.3	130	70.7	4568	69.4	
**Subgroup according to IHC using ER, PR and HER2**							
Hormone receptor+, HER2-	107	53.0	98	53.3	3390	51.5	0.385
Hormone receptor+, HER2-	27	13.4	18	9.8	916	13.9	
Hormone receptor-, HER2+	35	17.3	25	13.6	928	14.1	
Hormone receptor-, HER2-	33	16.3	43	23.4	1347	20.5	

HER2, human epidermal growth factor receptor-2; ER, estrogen receptor; PR, progesterone receptor; HbA1c, hemoglobin A1C

## Results

### Patient demographic and clinical characteristics

This study cohort comprised 6,581 nondiabetic patients, 202 diabetic patients receiving metformin, and 184 diabetic patients not receiving metformin. The demographic and clinical characteristics of the study patients are summarized in Table 1. Patients in the diabetic groups were older than those in the nondiabetic group (*P* <0.001), and the mean BMI levels were higher in the diabetic patient group (*P* <0.001). Patients with diabetes mellitus were also less likely to have received adjuvant chemotherapy than nondiabetic patients (*P* <0.001); however, there were no differences between the metformin and non-metformin groups (*P* = 0.155, by one-way ANOVA with post hoc Scheffé test). Tumor size, lymph node metastasis, ER and PR status, HER2-neu status, the percentages of patients receiving adjuvant hormone therapy, and the ratio of the four subgroups with ER, PR, and HER2-neu status analyzed by IHC were not significantly different among the three groups.

**Table 2:** Univariate Cox proportional hazards model for disease-free survival and cancer-specific survival

	Disease-free survival			Cancer-specific survival		
	*P*-value	Hazard ratio	95 % CI	*P*-value	Hazard ratio	95 % CI
**Metformin**						
Metformin		1			1	
Non-metformin versus metformin	0.05	1.50	1.0 to 2.25	0.02	1.69	1.07 to 2.68
Non-DM versus metformin	0.39	0.87	0.64 to 1.19	0.29	0.83	0.58 to 1.18
**Age, years**						
≤50	0.652	0.97	0.87 to 1.09	0.023	0.83	0.73 to 0.96
**BMI, kg/m2**						
High		1			1	
Low versus high	0.141	1.24	0.94 to 1.64	0.362	1.17	0.84 to 1.64
Normal versus high	0.132	0.91	0.80 to 1.03	0.064	0.87	0.75 to 1.01
**Tumor size, cm**						
T ≥2	<0.001	2.59	2.30 to 2.91	<0.001	3.10	2.68 to 3.59
**Node metastasis**						
Node-positive	<0.001	2.79	0.49 to 3.13	<0.001	3.58	3.10 to 4.13
**Estrogen receptor status**						
Positive	0.026	0.78	0.63 to 0.97	<0.001	1.85	1.62 to 2.11
**Progesterone receptor status**						
Positive	0.001	0.72	0.60 to 0.87	<0.001	1.93	1.69 to 2.21
**HER2 status**						
Positive	<0.001	1.44	1.28 to 1.62	<0.001	1.56	1.37 to 1.79
**Chemotherapy**						
Yes	0.098	0.82	0.65 to 1.04	<0.001	0.38	0.31 to 0.46
**Endocrine therapy**						
Yes	0.108	0.83	0.67 to 1.04	<0.001	1.78	1.56 to 2.03

HER2, human epidermal growth factor receptor-2; DM, diabetes mellitus

### Breast cancer survival according to diabetes mellitus and metformin treatment

**Fig. 4:**
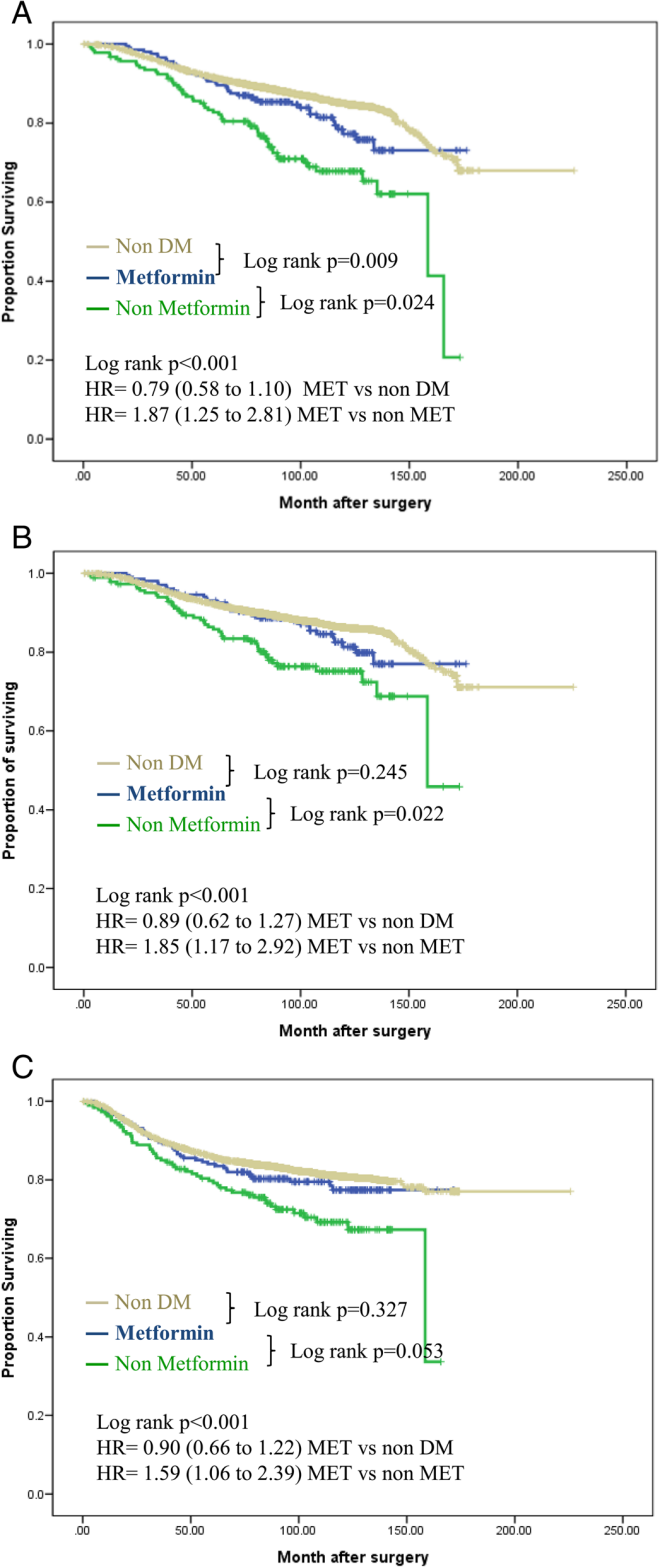
Breast cancer survival according to diabetes mellitus (DM) and metformin treatment (MET). **(a)** Overall survival. **(b)** Cancer-specific survival. **(c)** Disease-free survival. Adjusted for tumor size (≤2 versus > 2 cm), lymph node status (positive versus negative), estrogen receptor status, progesterone receptor status, human epidermal growth factor receptor-2 (HER2)-neu status (non-amplification versus amplification), chemotherapy and endocrine therapy

The median follow-up period was 100.3 months. In Kaplan-Meier analysis, the metformin group had significantly increased OS and cancer-specific survival compared with diabetic patients who did not receive metformin therapy (log-rank test, both *P* <0.005) (Fig. 4a, b). There were 1,262 recurrences (18.1 %): 42 (20.8 %) in the metformin group, 52 (28.3 %) in the non-metformin group and 1,168 (17.7 %) in the nondiabetic group. Patients in the metformin group experienced better DFS than diabetic patients in the non-metformin group, which was borderline statistically significant (log-rank test, *P* = 0.058; Fig. 4c) and there was no difference from the nondiabetic group. On univariate analysis, the HR for DFS in the non-metformin group compared with the metformin group was 1.50 (95 % CI 1.0 to 2.25), 1.69 (95 % CI, 1.07 to 2.68) for CSS (Table 2). After adjusting for age, BMI, tumor size, lymph node metastasis, ER, PR, and HER2-neu status, and systemic treatment, patients who did not receive treatment with metformin tended to have shorter OS (HR 1.87; 95 % CI 1.25 to 2.81), CSS (HR 1.85; 95 % CI 1.17 to 2.92) and DFS (HR 1.59; 95 % CI 1.06 to 2.39) than those of the metformin group (Fig. 4, Table 3).

**Table 3:** Multivariate Cox proportional hazards model for disease-free survival and cancer-specific survival

	Disease-free survival			Cancer-specific survival		
	*P*-value	Hazard ratio	95 % CI	*P*-value	Hazard ratio	95 % CI
**Metformin**						
Metformin		1			1	
Non-metformin versus metformin	0.025	1.59	1.06 to 2.39	0.009	1.85	1.17 to 2.92
Non-DM versus metformin	0.483	0.90	0.66 to 1.22	0.519	0.89	0.62 to 1.27
**Age, years**						
≤50	0.584	1.04	0.92 to 1.17	0.106	0.89	0.77 to 1.03
**Body mass index, kg/m2**						
High		1			1	
Low versus high	0.009	1.17	1.1 to 1.17	0.018	1.52	1.08 to 2.14
Normal versus high	0.639	1.03	0.91 to 1.17	0.597	1.04	0.90 to 1.21
**Tumor size, cm**						
T ≥2	<0.001	2.03	1.78 to 2.31	<0.001	2.18	1.86 to 2.56
**Node metastasis**						
Node positive	<0.001	2.59	2.26 to 2.96	<0.001	3.09	2.63 to 3.64
**Estrogen receptor status**						
Positive	0.009	0.79	0.66 to 0.94	0.026	0.78	0.63 to 0.97
**Progesterone receptor status**						
Positive	0.004	0.80	0.68 to 0.93	0.001	0.72	0.60 to 0.87
**HER2 status**						
Positive	<0.001	1.27	1.13 to 1.44	<0.001	1.29	1.12 to 1.49
**Chemotherapy**						
Yes	<0.001	0.70	0.58 to 0.84	0.098	0.82	0.65 to 1.04
**Endocrine therapy**						
Yes	0.312	0.91	0.75 to 1.10	0.108	0.83	0.67 to 1.04

HER2, human epidermal growth factor receptor-2; DM, diabestes mellitus

**Table 4:** Multivariate Cox proportional hazards model for disease-free survival by immunohistochemical staining of ER, PR and HER2

	Disease-free survival			Cancer-specific survival		
	*P*-value	Hazard ratio	95 % CI	*P*-value	Hazard ratio	95 % CI
**Hormone receptor-positive, HER2-negative**						
**Metformin**						
Metformin		1			1	
Non-metformin versus metformin	0.340	1.35	0.73 to 2.47	0.072	1.92	0.94 to 3.90
Non-DM versus metformin	0.484	0.85	0.54 to 1.34	0.64	0.87	0.50 to 1.54
**Age, years**						
≤50	0.369	0.92	0.76 to 1.12	0.012	0.728	0.57 to 0.93
**Body mass index, kg/m** ^**2**^						
High		1			1	
Low versus high	0.034	1.62	1.04 to 2.54	0.009	2.11	1.20 to 3.68
Normal versus high	0.772	0.97	0.80 to 1.18	0.87	0.98	0.76 to 1.26
**Tumor stage**						
T ≥2	<0.001	2.10	1.73 to 2.54	<0.001	2.44	1.88 to 3.16
**Node stage**						
Node-positive	<0.001	2.13	1.76 to 2.58	<0.001	2.92	2.25 to 3.80
**Hormone receptor-positive, HER2-positive**						
**Metformin**						
Metformin		1			1	
Non-metformin versus metformin	0.002	5.37	1.88 to 15.28	0.001	6.51	2.06 to 20.55
Non-DM versus metformin	0.648	1.23	0.50 to 3.02	0.502	1.41	0.52 to 3.84
**Age, years**						
≤50	0.120	0.79	0.59 to 1.06	0.006	0.623	0.45 to 0.87
**Body mass index, kg/m** ^**2**^						
High		1			1	
Low versus high	0.050	2.15	1.00 to 4.63	0.446	1.50	0.53 to 4.30
Normal versus high	0.053	1.41	1.00 to 1.99	0.085	1.41	0.95 to 2.09
**Tumor stage**						
T ≥2	<0.001	1.81	1.33 to 4.27	0.004	1.72	1.19 to 2.47
**Node stage**						
Node-positive	<0.001	2.22	1.64 to 3.02	0.000	2.85	1.97 to 4.13
**Hormone receptor-negative, HER2-positive**						
**Metformin**						
Metformin		1			1	
Non-metformin versus metformin	0.897	1.07	0.39 to 2.95	0.452	0.61	0.16 to 2.24
Non-DM versus metformin	0.310	0.72	0.38 to 1.36	0.156	0.61	0.31 to 1.21
**Age, years**						
≤50	0.310	1.17	0.89 to 1.54	0.385	1.15	0.84 to 1.57
**Body mass index, kg/m** ^**2**^						
High		1			1	
Low versus high	0.06	1.81	0.98 to 3.35	0.189	1.63	0.79 to 3.38
Normal versus high	0.615	0.93	0.68 to 1.25	0.471	0.88	0.63 to 1.24
**Tumor stage**						
T ≥2	<0.001	1.98	1.45 to 2.71	<0.001	2.39	1.63 to 3.51
**Node stage**						
Node-positive	<0.001	3.03	2.24 to 4.11	<0.001	3.94	2.71 to 5.72
**Hormone receptor-negative, HER2-negative**						
**Metformin**						
Metformin		1			1	
Non-metformin versus metformin	0.311	1.62	0.64 to 4.12	0.329	1.66	0.60 to 4.56
Non-DM versus metformin	0.89	1.05	0.50 to 2.24	0.867	1.07	0.47 to 2.42
**Age, years**						
≤50	0.350	1.13	0.88 to 1.44	0.989	1.00	0.76 to 1.32
**Body mass index, kg/m** ^**2**^						
High		1			1	
Low versus high	0.993	1.00	0.54 to 1.83	0.629	1.17	0.62 to 2.23
Normal versus high	0.750	1.04	0.81 to 1.34	0.583	1.08	0.82 to 1.43
**Tumor stage, cm**						
T ≥2	<0.001	1.62	1.26 to 2.08	<0.001	1.76	1.32 to 2.34
**Node stage**						
Node-positive	<0.001	2.25	1.78 to 2.84	<0.001	2.45	1.88 to 3.19

HER2, human epidermal growth factor receptor-2. DM, diabetes mellitus

### Subgroup analyses according to intrinsic subtype using immunohistochemical staining of ER, PR, and HER2

The Kaplan-Meier estimates of DFS stratified by the four subgroups are shown in Fig. 1. Hormone receptor-positive and HER2-positive patients showed a DFS benefit (log-rank test, *P* = 0.001; Fig. 1b) in those patients who received metformin compared with the diabetic non-metformin group, but the other subgroups showed no significant differences (Fig. 1a, c, d). Multivariate Cox proportional hazards regression analysis of the DFS of diabetic patients with hormone receptor-positive and HER2-positive breast cancer was carried out with a model consisting of the categorical covariates, age (<50 years versus ≥50 years), BMI (<25 kg/m^2^ versus ≥25 kg/m^2^), tumor size (T1 versus T2, 3, or 4), and node metastasis, using the metformin group as the reference. There were significant differences with regard to the risk of disease recurrence (HR 5.37; 95 % CI 1.88 to 15.28) and CSS (HR 6.51; 95 % CI 2.06 to 20.55) between the non-metformin and metformin groups, but there were no differences between the nondiabetic and metformin groups (Fig. 1b, Table 4).

### Subgroup analyses according to systemic treatment of breast cancer among nondiabetic patients, diabetic patients receiving metformin, and diabetic patients not receiving metformin

For patients who did not receive chemotherapy and/or endocrine therapy, there were no statistically significant differences in DFS among the nondiabetic, non-metformin, and metformin groups. (Fig. 2a, c, e). Among the patients who had received endocrine therapy only or chemotherapy only, the metformin treatment group did not differ in DFS compared with the non-metformin treatment group (Fig. 2f, g). Among the patients who received chemotherapy and endocrine therapy, the non-metformin diabetic group had decreased DFS (HR 2.14; 95 % CI 1.14 to 4.04) compared with the metformin group, after adjusting for age, BMI, tumor size, lymph node metastasis, ER, PR and HER2.

## Discussion

The results of this study confirmed that treatment with metformin after breast cancer diagnosis in diabetic patients improves DFS and reduces mortality. The most important finding was that the survival difference according to metformin treatment and diabetes was only observed on IHC staining in patients with hormone receptor-positive and HER2-positive tumors. The survival benefit was clear for patients who received chemotherapy or endocrine therapy compared with those who did not receive either of those treatments. These findings are consistent with several other recent analyses involving large numbers of subjects. To our knowledge, our present study is the first to include breast cancer clinicopathologic information and recurrence for the analysis of the relationship between metformin and breast cancer recurrence and mortality. The most common weaknesses of previous studies are a lack of information on the breast cancer characteristics, such as tumor size, lymph node metastasis, hormone receptor status, HER2 status, and treatment methods. These factors are the most important determinants of survival outcome. Hence, without considering the prognostic and predictive factors of breast cancer, any evaluation of the prognostic relationship between breast cancer and metformin is uncertain. Moreover, most previous studies have compared OS, and some have compared CSS, but few reports have explored the DFS in breast cancer and metformin treatment (Table 5) [12, 14, 15, 17–19].

**Table 5:** Literature reviews of metformin and breast cancer

Study	Year	Study population of breast cancer	Number (met/non-met/non-DM)	DFS	CSS	OS	Follow up (months or years)	HR (met versus non-met)^1^; 95 % CI	Special subgroup	Duration of metformin treatment	Tumor characteristics
He *et al*. [15]	2012	Stage II, HER2+ only	88/66/1829	N	Y	Y	47.6	OS (HR 0.52; 0.28 to 0.97)		NA	Done
								CSS (HR 0.47; 0.24 to 0.90)			
Bayraktar *et al*. [17]	2012	Triple-negative only	63/67/1318	Y	Y	Y	62	OS (HR 1.22; 0.66 to 2.28)		NA	Done
								RFS (HR 1.37; 0.78 to 2.40)			
								DMFS (HR 1.63; 0.87 to 3.06)			
Peeters *et al*. [12]	2013	All	508/550/NA	N	Y	Y		OS (HR 0.74; 0.58 to 0.96)	Increased risk in the first 12 months after discontinuation	Done	NA
								CSS (HR 0.88; 0.59 to 1.29)			
Lega *et al*. [14]	2013	All	1094/1267/NA	N	Y	Y	4.5 (years)	OS (HR 0.97; 0.92 to 1.02)		Done	NA
								CSS (HR 0.91; 0.81 to 1.03)			
Hou *et al*. [18]	2013	All	419/594/4612	N	N	Y	68	OS^2^ (HR 0.762; 0.6 to 0.986)		NA	Done
								OS^3^ (HR 1.708; 1.461 to 1.997)			
Xiao *et al*. [19]	2014	Luminal	275/405/5105	N	Y	Y	70	OS (HR 3.579; 1.506 to 8.506) luminal A		NA	Done
								OS (HR 3.232;1.839 to 5.678) luminal B (high Ki67)			
								OS (HR 2.034;1.019 to 4.059) Luminal B (HER2+)			
Kim *et al*. (present study)		All	202/184/6581	Y	Y	Y	100	OS (HR 1.87;1.25 to 2.81)	1.Hormone receptor+/HER2 +	NA	Done
								CSS (HR1.85;1.17 to 2.92)	2.Chemotherapy and endocrine therapy		
								DFS (HR 1.59, 1.06 to 2.39)			

^1^Hazard ratio (HR) for metformin (met) versus non-metformin (non-met) (reference): He *et al*., Peeters *et al*., Lega *et al*. HR for non-metformin versus metformin (reference): Bayraktar *et al*. Xiao *et al*., Present study

^2^Metformin versus non diabetes mellitus (DM) (reference) ^3^Non-metformin versus non diabetes mellitus (reference). OS, overall survival; CSS, cancer-specific survival; DMFS, distant metastasis-free survival; RFS, relapse-free survival; DFS, disease-free survival; HER2, human epidermal growth factor receptor-2

NA, Not applicable

Metformin is considered a hybrid anticancer compound that combines both long-lasting effects that involve the persistent lowering of blood insulin and glucose levels and the immediate potency of a cancer cell-targeting molecular agent that concurrently suppresses the pivotal AMPK/mTOR axis and several protein kinases, including crucial cancer-related tyrosine kinase receptors [20]. Metformin reduces circulating insulin levels in nondiabetic patients, which is relevant because higher insulin and C-peptide levels have been associated with poor outcomes in breast cancer patients. A window-o-opportunity study of metformin showed that it reduces Ki67 expression in breast cancer and increases apoptosis [13].

In our current study cohort, a beneficial effect of metformin was only evident in hormone receptor-positive and HER2-positive diabetic patients. Few studies have reported an effect of metformin in a specific subtype of breast cancer. Bayraktar *et al*. [17] reported that metformin use during adjuvant chemotherapy does not significantly impact survival outcomes in diabetic patients with triple-negative breast cancer. He *et al*. [15] demonstrated that metformin use was associated with significantly decreased HRs for breast cancer-specific mortality in diabetic women with HER2-positive breast cancer (HR 0.47; 95 % CI 0.24 to 0.90; *P* = 0.023).

The survival benefit of metformin for hormone-responsive and HER2-positive cancer can be explained via two mechanisms. First, metformin can decelerate the growth of hormone receptor-positive, HER2-positive breast cancer by suppressing the AKT/mTOR signaling pathway. HER2, insulin receptor, and IGF-I receptor all act through the same downstream signaling pathway via PI3K, AKT, and mTOR. Hence, type 2 diabetes mellitus can further accelerate the growth of HER2-positive breast cancer given that AKT/mTOR signaling is already active [15]. Zhu *et al*. [21] have revealed the development of metformin-related implications for breast cancer prevention by showing that its systemic administration selectively targets tumor-initiating cells in a clinically relevant prevention model. Interactions between PI3K/AKT/mTOR and the estrogen and growth factor signaling and receptor tyrosine kinase cascade occur at multiple levels to promote cell proliferation and survival [22, 23]. Therefore, metformin can show an anticancer effect through suppression of one or more of these signaling pathways, which is more significant for hormone receptor-positive and HER2-positive breast cancer.

As a second mechanistic explanation for the survival benefit of metformin in patients with hormone-responsive and HER2-positive breast cancer, metformin may increase the efficacy of systemic treatments for this subtype of tumors by overcoming resistance to these therapies. ER-positive and HER2-positive tumors are considered to be an endocrine-resistant subtype of breast cancer. The resistance to antihormonal treatment is due to activation of the mTOR pathway [24, 25] and hyperactivation of IGF-I receptor [26]. Crosstalk between ER and growth factor receptor pathways has been considered to be a cause for endocrine therapy resistance in breast cancer [27]. This resistance mechanism is important in diabetic patients because the activation of the insulin receptor and IGF-I receptor signaling pathway is the main mechanism of promoting cancer cell proliferation in patients with diabetes [28, 29]. The results of the BOLEROII study, which showed mTOR inhibitor efficacy for hormone-resistant metastatic breast cancer, have indicated that hyperactivation of PI3K/AKT/mTOR is the main mechanism of secondary antihormonal treatment resistance and that the combined inhibition of ER, HER2, and mTOR is an effective treatment [30, 31]. Metformin inhibits the PI3K/AKT/mTOR pathway through IGF receptor inhibition.

Questions remain, however, about the clinical benefits of metformin as an anticancer agent in patients with breast cancer. We have shown from our current analyses that survival differences according to diabetes and metformin administration were significant in patients who received chemotherapy or hormone therapy. This suggests that metformin may have therapeutic efficacy by cooperatively enhancing the potency of chemotherapy or hormonal therapy. Our current results are consistent with findings of previous studies that showed that diabetic patients receiving metformin and neoadjuvant chemotherapy have a higher pathologic complete response rate than diabetic patients not receiving this drug (odds ratio 2.95; 95 % CI 1.07 to 8.17; *P* = 0.04) [11]. Recently, a phase III trial of adjuvant metformin has been initiated in women with breast cancer (MCIC CTG MA32) [9]. However, several years of follow-up are needed to determine the survival benefits of metformin. Moreover, a phase II clinical trial evaluating the antitumor effect of neoadjuvant metformin in postmenopausal women with ER-positive breast cancer with letrozole plus metformin or placebo is ongoing [10]. This trial will likely indicate the antitumor effects of metformin in breast cancer.

The present study had several limitations. First, the small sample size limits our ability to make firm conclusions. Second, a limitation of the present study is the lack of information on the metformin dose. Patients often receive more than one antidiabetic medication or insulin and undergo changes in their pharmacotherapy regimen over time. A recent population-based study showed no significant association between cumulative duration of past metformin use and improved survival [14]. Moreover, because the present study focuses on metformin as an antitumor drug that can reduce tumor recurrence, the past history of metformin treatment before breast cancer diagnosis was not considered. Finally, this study did not consider the severity or duration of diabetes. The use of metformin in the diabetic cohort could result in better glucose control and help maintain adequate BMI. Diabetic patients who do not take metformin have increased comorbidity compared with patients who take metformin, indicating a possible selection bias. Serial reduction of BMI could not be evaluated in the present study. But this study compared serum glucose on breast cancer surgery and the hbA1C level, which stands for the severity of diabetes for diabetic patients, but there were no significant difference between the groups.

Both the tumor-suppressing activities of metformin and the tumor-promoting effects of other diabetic conditions may also contribute to the relative survival benefit of metformin observed in our current study. Our present findings support the hypothesis that metformin improves the survival of breast cancer patients with concurrent diabetes, particularly in cases with hormone-responsive and HER2-positive tumors, receiving adjuvant systemic therapy.

## Conclusion

This study is the first report to show an association between metformin and long-term breast cancer survival according to cancer subtype through the evaluation of breast cancer characteristics and metformin treatment. Moreover, our present findings can provide a background for future translational research and clinical work. We await the results of ongoing randomized trials of metformin as a treatment option for breast cancer patients.

## Abbreviations

AMCBCD: Asan Medical Center Breast Cancer Database
AMPK: adenosine monophosphate-activated protein kinase
ANOVA: analysis of variance
BMI: body mass index
CSS: cancer-specific survival
DFS: disease-free survival
ER: estrogen receptor
HER2: human epidermal growth factor receptor-2
HR: hazard ratio
IGF: insulin-like growth factor
IHC: immunohistochemical
mTOR: mammalian target of rapamycin
OS: overall survival
PR: progesterone receptor
